# Cardiac Magnetic Resonance Imaging and Arrhythmic Risk Stratification in Cardiomyopathies

**DOI:** 10.3390/jcm14144922

**Published:** 2025-07-11

**Authors:** Gianluca Di Bella, Antonino Micari, Roberto Licordari, Pasquale Crea, Luigi Colarusso, Maurizio Cusmà-Piccione, Rocco Donato, Tommaso D’Angelo, Giuseppe Dattilo, Antonino Recupero, Cesare de Gregorio, Antonio Micari, Giovanni Donato Aquaro

**Affiliations:** 1 Cardiology Unit, Department of Clinical and Experimental Medicine, University Hospital of Messina, 98124 Messina, Italy; gianluca.dibella@unime.it (G.D.B.);; 2 Department of Biomedical and Dental Sciences and Morphological and Functional Imaging, University of Messina, 98124 Messina, Italy; 3 Academic Radiology, Department of Surgical, Medical and Molecular Pathology and of Critical Area, University of Pisa, 56126 Pisa, Italy

**Keywords:** cardiomyopathies, cardiac magnetic resonance imaging, cardiac imaging, sudden cardiac death, ventricular arrhythmias

## Abstract

Cardiac magnetic resonance imaging (CMRI) has become an indispensable tool in evaluating arrhythmic risk and guiding therapeutic decisions in patients with non-ischemic cardiomyopathies (NICMs), including dilated (DCM), hypertrophic (HCM), and arrhythmogenic cardiomyopathies (ACM). Both European and American guidelines have given an additive and different value of late gadolinium enhancement (LGE) in specific morpho-functional (hypertrophic, dilated, and arrhythmogenic) phenotypes. In particular, LGE plays a different weight in relation to different cardiomyopathies. In dilated cardiomyopathy, LGE is able to predict arrhythmic risk in relationship to the presence and localization (septal and/or ring like LGE). On the contrary, in HCM, LGE is related to increased risk of cardiac death according to the extent (LGE >15%), while in ACM, it has a greater role in the presence of fat infiltration associated with LGE. In this review, we aim to identify predictors of sudden cardiac death related to myocardial structural features seen in CMRI in cardiomyopathies, going beyond the sole assessment of left ventricular function and ejection fraction.

## 1. Introduction

Cardiac magnetic resonance imaging (CMRI) has become an indispensable tool in evaluating arrhythmic risk and guiding therapeutic decisions in patients with non-ischemic cardiomyopathies (NICMs), including dilated cardiomyopathy (DCM), hypertrophic cardiomyopathy (HCM), and arrhythmogenic cardiomyopathy (ACM) [1,2]. NICMs are often associated with a higher risk of life-threatening arrhythmias, and accurately predicting arrhythmic risk is crucial for optimizing patient outcomes.

Cardiac magnetic resonance (CMR) excels in providing detailed, high-resolution images of cardiac structure and function, which are essential for understanding the pathophysiology of NICMs. In particular, CMR enables the assessment of myocardial fibrosis, a key predictor of arrhythmic events. Late gadolinium enhancement (LGE) on CMR identifies areas of myocardial scar and fibrosis, which are linked to abnormal electrical conduction and the substrate for arrhythmias. Quantifying the extent and distribution of fibrosis can help predict the likelihood of ventricular arrhythmias (VAs), including sudden cardiac death (SCD) [3].

Beyond risk stratification, CMR plays a crucial role in therapy decision making. In patients with high arrhythmic risk, CMR-guided identification of myocardial scarring can influence the decision to implant an implantable cardioverter–defibrillator (ICD) [4]. Moreover, it assists in monitoring disease progression, guiding medical therapy, and evaluating the effectiveness of interventions. In combination with other clinical data, CMR thus offers a comprehensive approach to managing NICMs, facilitating personalized treatment strategies aimed at improving long-term prognosis.

In addition to LGE, other CMR sequences like T1 and T2 mapping offer valuable insights into the pathophysiology of NICMs. T1 mapping quantifies myocardial tissue relaxation time, which can reflect interstitial fibrosis, edema, and inflammation. Elevated native T1 values are associated with myocardial fibrosis, providing a non-invasive way to assess fibrosis severity and track disease progression, particularly in conditions like HCM and DCM [5]. T2 mapping, on the other hand, is sensitive to myocardial edema and inflammation, making it useful in acute phases or in conditions like myocarditis [6]. Together, these advanced mapping techniques offer a comprehensive assessment of myocardial tissue composition, improving arrhythmic risk prediction and helping guide therapeutic strategies such as ICD implantation or antiarrhythmic therapy.

Recent European and American guidelines have clearly pointed out that the presence and/or extent of LGE is of primary importance in arrhythmic risk stratification [7,8]. The aim of this paper is to review the different roles of CMR and LGE in identifying arrhythmic risk in different cardiomyopathies.

## 2. Dilated Cardiomyopathy

DCM is a condition that affects the heart muscle, leading to enlargement and impaired function of the left ventricle (or both ventricles) without the presence of coronary artery disease or other factors that might cause volume or pressure overload, such as valvular, congenital, or hypertensive heart disease [9]. Advancements in medical treatment, SCD prevention with the use of ICDs, as well as the use of electric therapy such as cardiac resynchronization therapy (CRT) or cardiac contractility modulation (CCM) [10,11] have notably improved the survival rates for DCM patients. However, effectively predicting the risk of SCD in these patients remains a challenge. A key example is the DANISH Trial, which found that ICD implantation for primary prevention in patients with non-ischemic dilated cardiomyopathy (NI-DCM) did not reduce overall mortality, even though the result is not completely negative, since ICD implantation did lower the risk of SCD and increased the median age of death of the patients [12].

One of the main goals in the management of these patients is to correctly stratify their arrhythmic risk to identify the best candidates for ICD implantation. The European Society of Cardiology (ESC) guidelines still rely primarily on a left ventricular ejection fraction (EF) of 35% or lower as the main criterion for recommending ICD implantation in primary prevention [7]. However, according to a study by Pathak et al., up to 80% of patients with reduced EF and DCM never experience ventricular tachycardia (VT) or ventricular fibrillation (VF) during five years of follow-up [13]. For this reason, risk stratification based solely on ejection fraction value may lead to unnecessary implantation of many ICD devices.

**Figure 1:**
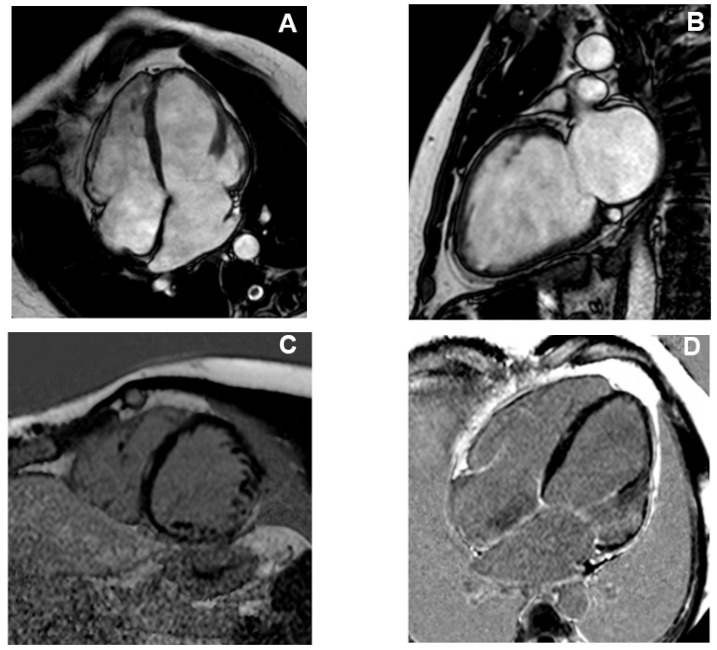
A patient with DCM and severe LV impairment (EF 25%) with a dilated left ventricle, as shown in balanced steady-state free precession imaging (bSSFP) in 4-chamber view (**A**) and 2-chamber view (**B**). LGE imaging in the short axis (**C**) and vertical long axis (**D**) shows the presence of LGE in the interventricular septum and the inferior and lateral walls. The patient developed an episode of VT, which was effectively terminated by the ICD (implanted following MRI).

Cardiovascular imaging can integrate the arrhythmic risk stratification of these patients thanks to the analysis of myocardial fibrosis (Figure 1). Indeed, the presence of LGE in the myocardium has been recognized as a potential trigger for ventricular arrhythmias [14,15], tripling the annual mortality risk and quintupling the risk of SCD/aborted-SCD compared with patients who did not present LGE [15]. For this reason, ESC guidelines consider the possibility of implanting a defibrillator in the presence of additional risk factors such as LGE in this type of patients [7].

Particularly, ESC guidelines have strongly emphasized that in patients with LVEF, >35% is needed to consider the presence of high-risk genes. In the absence of high-risk genes, the presence of a clinical findings of syncope and/or LGE in CMRI support the implantation of an ICD (class IIb) [7].

Metanalysis showed that even small amounts of LGE are associated with an increased risk of adverse events, in particular tachyarrhythmias and SCD [15,16]. The location of the LGE played a critical role in prognosis; in particular, the risk of SCD is high in the presence of scars of the interventricular septum or even higher when LGE is present in both the septal and lateral walls of the left ventricle [16].

Halliday BP et al. have shown that predictive models using LGE presence and location were superior to models based on LGE extent or pattern [17].

Nonetheless, a metanalysis by Di Marco et al. showed that LGE is strongly associated with ventricular arrhythmias and SCD, irrespective of their left ventricle ejection fraction (LVEF). For this reason, patients with DCM and LGE may benefit from ICD therapy, regardless of left ventricular function, whereas patients with left ventricular dysfunction but without LGE may not benefit [18].

Therefore, the assessment of myocardial fibrosis with CMR provides independent prognostic information beyond LVEF and should be carefully evaluated [19].

In DCM, the presence of septal LGE is associated with a large increase in the risk of death and SCD events, even when the extent is small. SCD risk is greatest with concomitant septal and free-wall LGE.

Moreover, the absence of fibrosis is associated with a reverse remodeling of the left ventricle, defined as an increase in ejection fraction and a reduction in end-diastolic diameter over time, whereas the increase in LGE extension during follow-up was associated with progressive LV dysfunction [20].

## 3. Hypertrophic Cardiomyopathy

Hypertrophic cardiomyopathy (HCM) is an inherited disease of the heart muscle characterized by increased left ventricular wall thickness that occurs without abnormal loading conditions. This condition affects approximately 1 in 500 people in the general population. SCD, which is caused by ventricular arrhythmias, occurs in about 1% of patients with HCM [7,8,21,22] and can sometimes be the first manifestation of the disease [23].

Both European (ESC) [7] and American (AHA/ACC) [8] guidelines agree on recommending ICD implantation for people who have experienced prior event such as resuscitated SCD, sustained VT or VF. However, for the remaining population, it is mandatory to stratify the arrhythmic risk to avoid unnecessary ICD implantation and related complications. With the widespread adoption of ICDs, physicians have worked to identify the most suitable candidates for primary prevention ICD implantation, leading to the development of risk stratification models for HCM patients.

Traditional risk factors include family history of SCD at young age; syncope, in particular non-neuromodulated syncopal episodes [7]; maximum LV thickness (studies have identified LV mass thickness as an independent risk factor for SCD, particularly when septal thickness exceeds 30 mm on echocardiographic measurements) [24,25]; and non-sustained ventricular tachycardia (NSVT) episodes [26,27].

Age plays also an important role; indeed, SCD occurs predominantly in younger patients, with the risk decreasing after the sixth decade [22,28]. In children, SCD risk is very low below age six [29], with a peak between ages nine and fifteen [30].

Left ventricular outflow tract obstruction (LVOTO), which is present in approximately two-thirds of HCM patients, has been associated with increased mortality from various causes, including SCD [31]. Promising new treatments for this condition may provide clinical benefit to patients suffering from it [7].

LV systolic dysfunction and apical aneurysms show a positive correlation with SCD [32,33,34]. ESC guidelines recommend considering these variables in shared decision making for ICD implantation [7].

**Figure 2:**
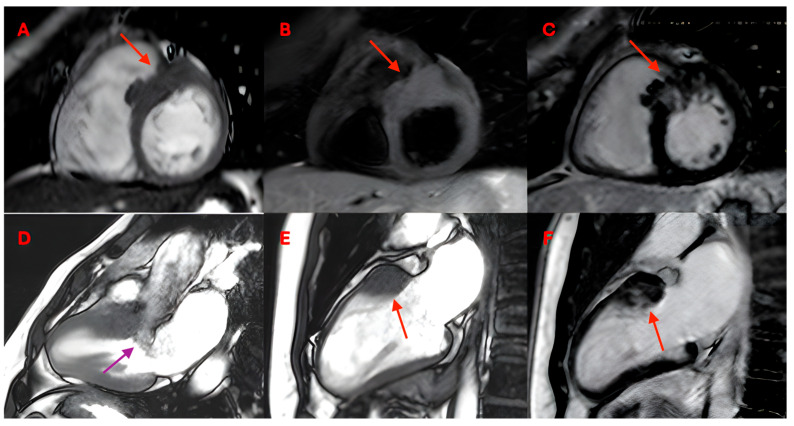
A 37-year-old patient with hypertrophic cardiomyopathy. (**A**) shows a short-axis bSSFP image in end diastole with focal thickening of the mid-anterior interventricular septum and mid-anterior wall. (**B**) shows a short-axis short tau inversion recovery (STIR) image demonstrating thickening of the same region without evidence of myocardial edema. In (**C**) is a short-axis LGE image showing mid-wall fibrosis in the mid-anterior wall and anterior septum (red arrows). (**D**) shows a three-chamber bSSFP image in end systole demonstrating LVOT obstruction with systolic anterior motion (SAM) of the anterior mitral leaflet and associated dynamic flow acceleration (purple arrow). (**E**) is a two-chamber bSSFP in end diastole showing focal basal anterior wall hypertrophy (red arrow). (**F**) reports a two-chamber LGE image depicting mid-wall late enhancement in the basal anterior wall (red arrow).

Advanced cardiovascular imaging may provide useful prognostic information for patients with HCM. Indeed, CMRI makes possible to assess the phenotype of HCM, allowing for the study of morphological and structural anomalies that are difficult to recognize with other imaging methods (Figure 2). Moreover, it has been shown to be an imaging technique with a central role in the assessment of hypertrophic phenotype and its diagnostic and prognostic implications [2].

Moreover, myocardial fibrosis, as measured by LGE on CMRI, is a predictor of arrhythmic events (VT or VF), as shown in a perspective study on 217 HCM patients by O’Hanlon et al. [35]. The risk of SCD is related to the LGE extent in LV myocardium: an LGE of ≥15% of LV mass demonstrated a 2-fold increase in SCD event risk in the patients otherwise considered to be at lower risk in a cohort of 1293 HCM patients [36].

Also, the progression of LGE in consecutive CMR examinations is related to a worsening of the clinical condition of HCM patients; it has been shown to be more extensive in apical hypertrophy than in other patterns [37], and it is correlated to the reduction of left ventricular systolic function and an increase in heart failure admission [38].

In addition to the extent of LGE at CMRI (≥15%), importance was also given to the dispersion of LGE within the muscle to quantify the risk of SCD: a higher dispersion provides a better risk stratification compared to the presence of LGE alone [39].

For these reasons, ESC recommends CMRI evaluation in patients fulfilling diagnostic criteria for HCM, after initial risk screening, especially in patients with suspected apical hypertrophy or aneurysm [40].

The ESC guidelines recommend using a multivariate scoring system for HCM-related SCD risk assessment to decide whether to implant a defibrillator. It is based on echocardiographic, clinical, and electrocardiographic variables, providing a result that estimates the 5-year risk of SCD. LGE assessment with CMRI is not evaluated in the first instance, but it has a central role in deciding whether to implant a defibrillator in patients with a low risk profile (<4%), along with the presence of apical aneurysm or the reduction of LVEF (<50%) [7].

Therefore, an individualized patient evaluation supported by risk scoring systems remains crucial for determining the optimal therapeutic approach. This personalized assessment ensures that each patient receives the most appropriate treatment strategy based on their specific risk profile.

## 4. Arrhythmogenic Cardiomyopathy

Arrhythmogenic cardiomyopathy (ACM) is a heart disease characterized by a fibrotic and/or fibrofatty replacement of myocardial tissue and a consequent predisposition to ventricular arrhythmic events with a higher susceptibility to SCD, especially in the young. The structural alterations underlying cardiomyopathy (scarring and fibrofatty replacement) are the substrate of arrhythmic events [7,41].

A genetic testing in patients with ACM is fundamental for the clinical management of affected patients, particularly for arrhythmic risk stratification. The most involved genetic mutations affect desmosomal proteins, such as desmocollin-2 (DSC2], plakophilin-2 (PKP2], plakoglobin (JUP), desmoplakin (DSP), and desmoglein-2 (DSG2]. In phenotypes with biventricular or left ventricular involvement, mutations in non-desmosomal proteins—including lamin A/C (LMNA/C), desmin (DES), N-cadherin (CDH2], and filamin C (FLNC)—are considered to be strongly causative. Worse prognosis and earlier symptoms were found to be related to carriers of more than one mutation (digenic or compound heterozygous variants) [42].

Given this risk, determining whether an ICD is necessary becomes a pivotal clinical decision after diagnosis. This is particularly important, as ACM frequently affects younger individuals, and SCD may be the initial sign of the condition.

For this reason, different algorithms for the calculation of the risk of SCD in patients with ACM were elaborated in this last decade. One of the largest studies was conducted by Cadrin-Tourigny et al. in 2019 [43], with the purpose to create a prediction model for ventricular arrhythmias in ACM patients. This algorithm is based on eight clinical predictors: sex, age, recent (<6 months) cardiac syncope, NSVT, number of PVCs on 24 h Holter monitoring, extent of T-wave inversion (TWI) on anterior and inferior leads, right ventricle (RV) ejection fraction, and left ventricle ejection fraction. This model allows for reducing the number of inappropriate ICD implantations but showed a limitation, that is, the use of ICD shocks as a surrogate of SCD; a great proportion of arrhythmias in ACM patients would have been self-limited in the absence of ICD therapy [43].

**Figure 3:**
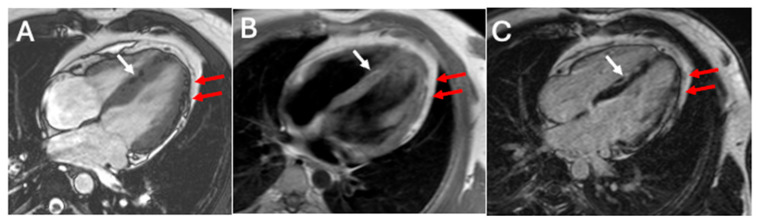
bSSFP imaging on four-chamber view shows India ink artifacts in left ventricular anterolateral wall (red arrows) and in inferior septum (white arrow), suggesting intramyocardial fat (**A**); T1 imaging (**B**) shows the presence of fibrofatty replacement (red arrows on inferolateral and white arrow on septal wall); LGE is observed in the same left ventricular areas (**C**), confirming the presence of fibrofatty LV damage and suggesting left-dominant ACM.

The diagnosis of ACM must rely on diagnostic criteria. Those proposed by the international task force in 2010 [44] lacked tissue characterization via CMRI and did not allow for the diagnosis of left-sided variants of the disease. This led to the development of the Padua criteria in 2020, which included CMR-based assessment of LGE to detect fibrofatty infiltration, allowing the final diagnosis of dominant-right, biventricular, or dominant-left ACM [45]. A CMRI report of a case of ACM is showed in Figure 3.

LGE in ACM plays a pivotal role in defining the prognosis of the patients. Aquaro et al. indicated that ACM cases with LV involvement (dominant-left or biventricular) have a poorer prognosis compared to isolated right ventricular forms. These patients face a higher risk of SCD, aborted cardiac arrests, and appropriate ICD shocks. Therefore, LV involvement alone is an independent predictor of major events, and the possibility of implanting an ICD should be evaluated [46,47].

The distribution of LGE in ACM patients provides another useful prognostic information, as shown by Yang et al. Indeed, ringlike LGE provides independent and incremental prognostic value over the 2019 ARVC risk model in predicting sustained VA in patients with ACM [48].

## 5. Emerging CMR Parameters

Recent advancements in CMR techniques—particularly T1, T2, and extracellular volume (ECV) mapping, feature tracking (FT), and scar heterogeneity evaluation—have significantly enhanced myocardial tissue characterization. By allowing a more precise and in-depth assessment of myocardial tissue and function, these techniques are increasingly recognized for their role in arrhythmic risk stratification in cardiomyopathies.

In HCM, several recent studies suggest that CMR mapping parameters offer added prognostic value in stratifying arrhythmic risk. For instance, Qin et al. conducted a prospective study involving 203 HCM patients and found that elevated native T1 values were independently associated with major adverse cardiovascular events (MACEs) during follow-up, even in low-risk patients [49]. A growing body of research has focused on ECV mapping, which quantifies interstitial fibrosis by combining pre- and post-contrast T1 mapping data. In a study of 73 patients with HCM [50], global ECV was the best parameter with which to identify patients with a risk of SCD ≥ 4% and patients with syncope or NSVT at the follow-up. Specifically, an ECV >34% correlated with a history of syncope or NSVT and a five-year SCD risk ≥4%, according to the HCM Risk-SCD model. The area under the curve (AUC) for ECV in predicting high-risk patients was ~0.83, better than LGE. Adding ECV to the standard SCD risk score improved prognostic accuracy for identifying candidates for ICD implantation. In a study of 108 HCM patients followed over two years, Yu et al. showed that global ECV was the best predictor of SCD, outperforming both LGE and native T1 in identifying high-risk individuals [51].

Beyond fibrosis, myocardial edema assessed via T2 mapping also appears prognostically relevant in HCM. Xu et al. followed approximately 700 HCM patients for ~3 years and found that elevated T2 values were associated with an increased risk of adverse outcomes. Patients with both LGE and high T2 values exhibited higher incidence of the composite endpoint of cardiac death and appropriate ICD shocks [52].

Feature tracking-derived strain metrics have also shown strong associations with arrhythmic events in HCM. For example, Barbosa et al. studied 109 HCM patients and found those with documented VAs/SCD (26% of cohort) had significantly worse GLS and GCS values than those without arrhythmias. In that study, the mean GLS was −13.2% in patients with VAs vs. −15.5% in those without (*p* = 0.011), and the GCS was −15.3% vs. −18.3% (*p* = 0.017). Additionally, patients deemed high-risk by SCD risk scores also showed more impaired strain (e.g., GLS ~−12.6% in high-risk vs. −15.5% in others) [53]. The multicenter STRAIN-HCM study (130 HCM patients with 5-year SCD risk <6% according to HCM Risk-SCD score) demonstrated that CMR GLS adds independent prognostic value. Over ~4.3 years, the composite endpoint (SCD, resuscitated VT/VF, or heart-failure hospitalization) occurred in 40 patients. On multivariate Cox analysis, GLS remained an independent predictor of outcome (HR ~1.12 per 1% strain worsening) even after adjusting for conventional risk factors like LVEF and LGE [54]. On the contrary, in a Hungarian cohort of 187 HCM patients, CMR strain metrics correlated with the degree of hypertrophy and LGE, but in that study, only LV mass index and LGE extent (not strain) significantly predicted pure arrhythmic outcomes on multivariate analysis [55].

Recent studies have increasingly explored the prognostic value of scar heterogeneity in stratifying arrhythmic risk across cardiomyopathies. Among these methods, the Global Dispersion Score (GDS) has emerged as a promising metric that quantifies the degree of LGE signal heterogeneity by assessing the pixel-level variability in signal intensity surrounding each LGE voxel. Greater dispersion may reflect a more complex arrhythmogenic substrate, thereby improving risk prediction over the mere presence or extent of fibrosis. In a 2020 study by Aquaro et al., GDS was shown to independently predict SCD and ventricular tachyarrhythmias in 183 HCM patients with low-to-intermediate risk by conventional criteria. A GDS threshold of 0.86 identified patients with a nearly tenfold increase in arrhythmic events. GDS outperformed conventional fibrosis measures: it reclassified risk beyond LGE scar extent >15% of LV mass (the guideline threshold) [39]. These findings were further substantiated in a large multicenter HCM cohort (n = 1229) to show that radiomic quantification of fibrosis heterogeneity significantly enhanced SCD risk stratification. A model using three principal radiomic features achieved a c-statistic of 0.69, outperforming the ESC five-year risk score (c 0.57, *p* = 0.02). Combining radiomics with the clinical models raised c-statistics to ~0.73–0.76 and achieved a net reclassification improvement of ~0.25–0.39 over the ESC model, particularly when integrated with existing clinical risk scores [56].

In DCM, risk stratification has traditionally relied on left ventricular ejection fraction (LVEF ≤ 35%) to identify ICD candidates. CMR mapping techniques offers more direct indicators of tissue remodeling. A recent multicenter prospective study involving 225 non-ischemic DCM patients demonstrated that both native T1 and global ECV were independently associated with arrhythmic events over a 2-year follow-up. Patients with T1 Z-scores > 4.2 and ECV > 30.5% had a 2.7-fold increased risk of arrhythmias [57]. Similarly, Li et al. showed in a cohort of 659 DCM patients that elevated native T1 and ECV values had significant prognostic value, especially in the absence of LGE [58]. Rubís et al. further demonstrated that global and segmental ECV values were significantly higher in DCM patients with a high arrhythmic burden (frequent NSVT or documented VT), even when LGE was similar across groups. ECV emerged as an independent predictor of arrhythmias: a 1% increase in ECV was associated with a ~12% relative risk increases for ventricular arrhythmias [59]. In DCM patients, T2 mapping values could be elevated, reflecting an underlying inflammatory process [60]. However, current evidence does not clarify whether these alterations are associated with an increased arrhythmic risk.

CMR feature tracking in DCM has also proven to be a strong prognostic tool. Tan et al. analyzed 364 patients with DCM who underwent primary or secondary prevention ICD implantation and a CMR pre-implant. Over ~5 years, 118 patients (32%) met the composite endpoint of appropriate ICD shock or death. CMR GLS proved to be an independent predictor of outcomes: in multivariate analysis including LVEF, a 1% less negative GLS was associated with a 5% higher hazard of death or shock (HR 1.05 per 1%; *p* = 0.01). Notably, the predictive value of GLS remained robust regardless of ischemic vs. non-ischemic etiology or baseline EF. This suggests that even among already-indicated ICD patients (most with EF < 35%), strain can further stratify risk of appropriate therapies [61]. There is growing interest in the right ventricular function in DCM. A 2021 multicenter study of 273 patients with idiopathic DCM found that RV free-wall longitudinal strain by CMR was a prognostic indicator. Over 3+ years, 15% of patients experienced major adverse cardiac events, including life-threatening ventricular arrhythmias or ICD interventions. RV-GLS, together with LGE, emerged as the strongest CMR predictors of outcome. Adding RV-GLS to a model with standard predictors significantly improved prognostic accuracy (AUC improved from 0.71 to 0.76, *p* = 0.03) [62]. LV GLS remains the primary strain metric for risk stratification in DCM, but assessing RV strain (and left atrial function) may further refine risk, especially for ventricular arrhythmias and SCD.

In patients with DCM, the GDS method has been most rigorously validated. Aquaro et al. demonstrated, in a cohort of 510 DCM patients, that a GDS value greater than 0.10 was strongly associated with malignant ventricular arrhythmias, including SCD and appropriate ICD interventions. This prognostic significance remained independent of LVEF and total LGE burden. Notably, GDS was predictive across a wide spectrum of LVEFs, including patients traditionally considered lower risk [63]. These findings were complemented by Amyar et al., who applied radiomic texture analysis to LGE-CMR (extracting features like gray-level co-occurrence matrix (GLCM) metrics) in a separate DCM population. Three key radiomic features were identified; adding these LGE texture metrics to clinical + scar models significantly improved arrhythmia risk prediction (C-statistic rose from ~0.61 to ~0.70 in both development and validation cohorts, *p*≈0.03]. One texture feature—GLCM autocorrelation—emerged as an independent predictor of VT/VF (HR 2.4 in external validation) [64].

In ACM, risk stratification remains imperfect. CMR mapping in ACM is technically challenging due to the thin RV wall and potential fat infiltration. Some studies have evaluated LV mapping in ACM. Chun et al. found that elevated LV T1 and ECV values correlated with increased risk of heart failure events but not with arrhythmic outcomes [65]. However, a more recent study involving 91 ACM patients followed over 4.5 years showed that reduced biventricular global longitudinal strain (GLS) and elevated LV ECV were independently associated with sustained ventricular tachyarrhythmias. For each 1% worsening in GLS of the left or right ventricle, the risk of arrhythmia increased by approximately 14% and 9%, respectively. Similarly, each 1% increase in left ventricular ECV was associated with a ~13% higher arrhythmic risk (HR ~1.13; *p* < 0.001) after adjustment for the standard ARVC risk score. Moreover, adding biventricular GLS and ECV to the clinical model significantly improved risk stratification: for example, the AUC increased from ~0.65 with the ARVC risk score alone to ~0.73 when strain and ECV were included (*p* < 0.001) [66]. Another study highlighted that left ventricular longitudinal dyssynchrony (LVLD) ≥ 89.15 ms, as measured by FT-CMR, was an independent predictor of cardiovascular and arrhythmic events. However, in patients with advanced right ventricular dysfunction, the prognostic value of LV GLS and diastolic strain rate (DSR) appeared less consistent [67].

By enabling quantitative assessment of myocardial fibrosis, edema, deformation, and scar complexity, techniques such as mapping, feature tracking, and LGE heterogeneity analysis offer valuable prognostic insights that go beyond traditional measures like LVEF or LGE presence alone. As evidence continues to accumulate, the integration of these parameters into clinical decision-making algorithms may enhance the identification of high-risk patients and optimize the selection of candidates for prophylactic ICD implantation.

## 6. Conclusions

CMRI has emerged as a pivotal imaging technique in the arrhythmic risk stratification of cardiomyopathies, offering superior tissue characterization, accurate assessment of ventricular volumes and function, and detailed visualization of myocardial fibrosis through LGE. The presence, extent, and distribution of fibrosis identified by CMR have shown strong associations with ventricular arrhythmias and SCD across a range of cardiomyopathies, including DCM, HCM, and ACM. Indeed, in DCM, the evaluation of arrhythmic risk cannot be based only on the ejection fraction but must also consider the presence of LGE. In HCM, the extent of LGE significantly alters the prognosis, with a higher risk of SCD in case of LGE > 15%. Finally, also in ACM, CMRI plays a pivotal role in the diagnosis of the disease and in the definition of the prognosis of the patients.

Moreover, new emerging techniques—including native T1, ECV, T2 mapping, and feature tracking strain analysis—can significantly enhance arrhythmic risk stratification across these cardiomyopathies. In HCM, elevated ECV, T2 values, and impaired GLS consistently correlate with higher arrhythmic risk, even in patients with low-to-intermediate conventional risk scores. In DCM, native T1, ECV, and CMR-derived strain metrics, particularly LV and RV GLS, serve as independent prognostic markers, regardless of LVEF. In ACM, although technical challenges persist, emerging evidence supports the prognostic role of biventricular GLS, LV ECV, and strain-based dyssynchrony indices.

Overall, CMRI offer powerful tools to refine individual risk assessment, and the integration of imaging findings with available risk models can potentially allow for more accurate selection for ICD implantation, improving the clinical outcomes and patient prognosis.

## 7. Future Directions

With advancements in imaging techniques and tissue characterization, particularly with CMR and cardiac computed tomography, it will be easier to precisely detect myocardial fibrosis and other structural abnormalities associated with arrhythmogenesis. The integration between morphological data from imaging and the use of novel biomarkers will allow doctors to better select who may benefit from ICD therapy. Furthermore, the incorporation of artificial intelligence and machine learning algorithms promises to enhance the diagnostic value of CMRI by analyzing complex imaging patterns in large datasets. As evidence grows, guidelines are likely to evolve, promoting a more central role for CMRI in tailoring arrhythmic risk assessment and guiding therapy in patients with various forms of cardiomyopathy.

## Data Availability

Not applicable.

## Abbreviations

The following abbreviations are used in this manuscript:

CMRI	cardiac magnetic resonance imaging
NICMs	non-ischemic cardiomyopathies
DCM	dilated cardiomyopathy
HCM	hypertrophic cardiomyopathy
ACM	arrhythmogenic cardiomyopathy
LGE	late gadolinium enhancement
ICD	implantable cardioverter–defibrillator
CRT	cardiac resynchronization therapy
SCD	sudden cardiac death
LVEF	left ventricle ejection fraction
